# Selective Thyroid Hormone Receptor-Beta (TRβ) Agonists: New Perspectives for the Treatment of Metabolic and Neurodegenerative Disorders

**DOI:** 10.3389/fmed.2020.00331

**Published:** 2020-07-09

**Authors:** Federica Saponaro, Simona Sestito, Massimiliano Runfola, Simona Rapposelli, Grazia Chiellini

**Affiliations:** ^1^Department of Pathology, University of Pisa, Pisa, Italy; ^2^Department of Pharmacy, University of Pisa, Pisa, Italy; ^3^Interdepartmental Research Centre for Biology and Pathology of Aging, University of Pisa, Pisa, Italy

**Keywords:** selective thyromimetics, prodrugs, thyroid hormone receptors, dyslipidemia, non-alcoholic steatohepatitis (NASH), non-alcoholic fatty liver disease (NAFLD), myelin, clinical trials

## Abstract

Thyroid hormones (THs) elicit significant effects on numerous physiological processes, such as growth, development, and metabolism. A lack of thyroid hormones is not compatible with normal health. Most THs effects are mediated by two different thyroid hormone receptor (TR) isoforms, namely TRα and TRβ, with the TRβ isoform known to be responsible for the main beneficial effects of TH on liver. In brain, despite the crucial role of TRα isoform in neuronal development, TRβ has been proposed to play a role in the remyelination processes. Consequently, over the past two decades, much effort has been applied in developing thyroid hormone analogs capable of uncoupling beneficial actions on liver (triglyceride and cholesterol lowering) and central nervous system (CNS) (oligodendrocyte proliferation) from deleterious effects on the heart, muscle and bone. Sobetirome (GC-1) and subsequently Eprotirome (KB2115) were the first examples of TRβ selective thyromimetics, with Sobetirome differing from the structure of thyronines because of the absence of halogens, biaryl ether oxygen, and amino-acidic side chain. Even though both thyromimetics showed encouraging actions against hypercholesterolemia, non-alcoholic steatohepatitis (NASH) and in the stimulation of hepatocytes proliferation, they were stopped after Phase 1 and Phase 2–3 clinical trials, respectively. In recent years, advances in molecular and structural biology have facilitated the design of new selective thyroid hormone mimetics that exhibit TR isoform-selective binding, and/or liver- and tissue-selective uptake, with Resmetirom (MGL-3196) and Hep-Direct prodrug VK2809 (MB07811) probably representing two of the most promising lipid lowering agents, currently under phase 2–3 clinical trials. More recently the application of a comprehensive panel of ADME-Toxicity assays enabled the selection of novel thyromimetic IS25 and its prodrug TG68, as very powerful lipid lowering agents both *in vitro* and *in vivo*. In addition to dyslipidemia and other liver pathologies, THs analogs could also be of value for the treatment of neurodegenerative diseases, such as multiple sclerosis (MS). Sob-AM2, a CNS- selective prodrug of Sobetirome has been shown to promote significant myelin repair in the brain and spinal cord of mouse demyelinating models and it is rapidly moving into clinical trials in humans. Taken together all these findings support the great potential of selective thyromimetics in targeting a large variety of human pathologies characterized by altered metabolism and/or cellular differentiation.

## Introduction

**Figure 1:**
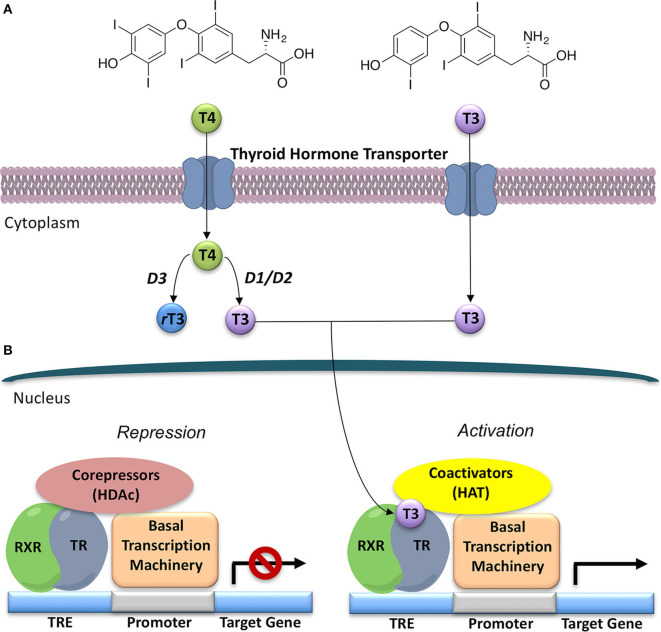
General model for thyroid hormone (T3) generation and action in the nucleus. **(A)** Thyroxine (T4), the prevalent form of thyroid hormone (TH) produced by the thyroid gland, once released is converted to T3, the major form of TH, by deiodinases 1 and 2 (D1 and D2). Deiodinase 3 (D3) converts T4 to the inactive rT3. **(B)** Thyroid Hormone Receptors (TRs) act as transcriptional factors that regulate a wide variety of genes. Unliganded TR, often as a heterodimer with retinoic X receptor (RXR), binds to a thyroid hormone response element (TRE) on DNA, and then recruits co-repressors and their associated histone de-acetylases (HDACs); thus, repressing gene expression. T3 binding to the ligand-binding domain (LBD) promotes a conformational change in TR that leads to co-repressor release and recruitment of co-activators, and associated histone acetyl-transferases (HATs). This alteration in co-factor binding leads to recruitment of polymerase III and the onset of gene transcription.

Thyroid hormones (THs), namely 3,3′,5,-triiodo-L-thyronine (T3), 3,3′,5,5′-tetraiodo-L-thyronine (T4) and their metabolites refer to a group of tyrosine-based molecules rich in iodine, which exert a key regulatory role in human metabolism (1, 2). Indeed, THs are involved in fetal tissues differentiation, brain development, cardiovascular homeostasis, skeletal maintenance, and in the control of carbohydrates and lipids metabolism (3–5). Thyroxine (T4) is the prevalent form of TH, produced and released from the thyroid gland in humans. Once released, T4 is converted into T3, the major form of TH, by two enzymes called deiodinases type I (D1) and type II (D2), whereas type III deiodinases (D3) leads to the formation of reverse T3 (rT3), that represents a key step in the process of TH inactivation [(2); Figure 1]. This classical picture in the last decade turned to be simplistic, since different classes of enzymes, namely deiodinases, amine transferases, amine oxidases, decarboxylase, and sulfotransferases, have been demonstrated to act on T4 and T3. As a matter of facts, several derivative metabolites of T4 and T3, active on THs receptors, are produced and identified as novel thyroid hormones. These are 3,5-didiodothyronine (T2); thyronamines, particularly 3-iodothyronamine (T1AM) and non-iodinated thyronamines (T0AM); thyroacetic acids, particularly 3,5,3′,5′-thyroacetic acid (TA4), 3,5,3′-thyroacetic acid (TA3), 3-thyroacetic acid (TA1), which will be not discussed in details in this review (6, 7).

During the past few decades, increasing interest has been directed toward a possible therapeutic use of THs and their analogs in the field of dyslipidemia and liver diseases, with an abundance of data coming from many new promising compounds (8–10). Initially the idea that thyromimetics could be useful in lipid metabolism came from the clinical observation that patients with hyperthyroidism show an important reduction of body weight and serum cholesterol. Unfortunately, they also suffer from many unwanted secondary effects such as cardiovascular impairment, muscle and bone loss and depression (11). Thus, the potential to uncouple the beneficial effects of THs from side effects offers an intriguing challenge.

**Figure 2:**
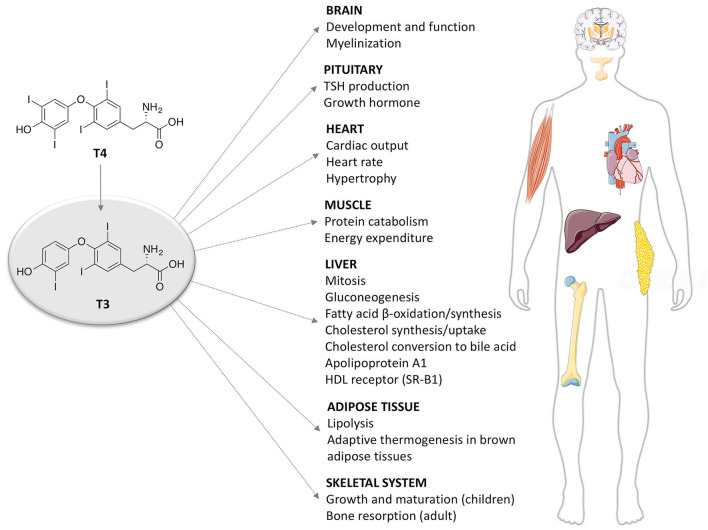
Summary of thyroid hormone (T3) effects on different tissues.

THs actions (Figure 2) can be mediated by THs receptors (TRs) or not, leading to a wide range of effects, previously described as genomic and non-genomic effects (12, 13). Recently a revision of this over simplified description has been proposed with a novel classification of THs effects according to the involved molecular pathways (14), as reported in the next paragraph. THs receptors (TRs) have been known since 1986 (15, 16). They are part of a larger family of intracellular receptors that includes those for steroid hormones, retinoic acid, vitamin D, and peroxisomal proliferators (17). TRs act as transcriptional factors that regulate a wide variety of genes through the interaction with specific co-activators, co-repressors and DNA sequences (TH response elements—TREs), located in the regulatory regions of target genes [(2, 18, 19); Figure 1].

**Figure 3:**
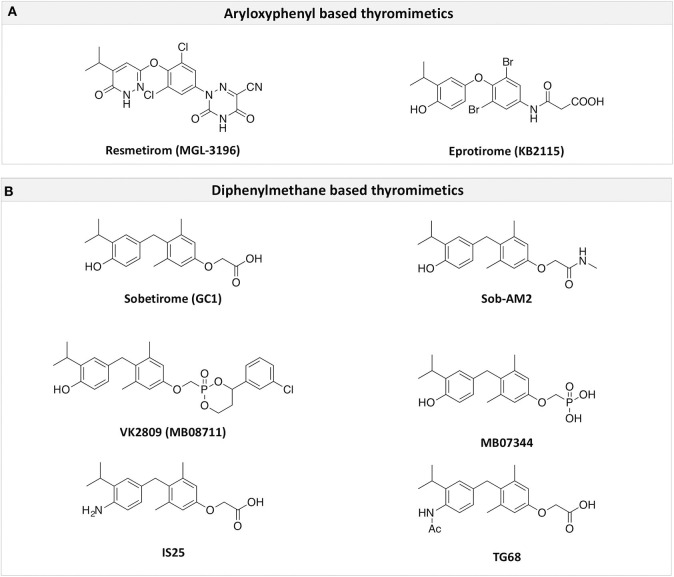
A structure-based representation of TRβ-selective thyromimetics. **(A)** AryloxyPhenyl based thyromimetics include: Resmetirom (MGL-3196) and Eprotirome (KB2115). **(B)** Diphenylmethane based thyromimetics include: Sobetirome (GC1), Sob-AM2, VK2809 (MB08711), MB07344, and novel IS25 and TG68.

The two isoforms TRα and TRβ are encoded by two genes, *THRA* on chromosome 17 and *THRB* on chromosome 3 in human species (*Thra* and *Thrb* in mice), respectively, and various forms of TR proteins are produced, through alternative splicing (20). The distribution of specific isoforms varies between different tissues and species: in human, TRα is proved to be predominant in the heart, brain and bone, whereas TRβ is prevalent in the liver, kidneys, pituitary gland, and brain, and it is responsible for THs effects on metabolism (21–23). Based on this consideration, in the last few decades considerable effort has been devoted to developing compounds endowed of selectivity for the beta isoform of the TRs, that would be effective on the treatment of metabolic and/or on brain disorders, without producing deleterious effects on heart and bone. To date, several molecules have been developed (Figure 3), including Sobetirome (GC-1) and Eprotirome (KB2115), just to mention two compounds that have been extensively investigated for many years. These molecules showed great promise of becoming drugs for the treatment of lipid metabolism disorders, but ultimately failed to reach the therapeutic market because of the onset of unwanted side-effects (23). Noteworthy, in recent years there has been a resurgence of interest in developing TRβ selective thyromimetics, and in particular “tissue-selective prodrugs” able to release the desired active compound at the site of action (24). The aim of the present review is to provide an update regarding the most significant steps toward the obtainment of clinically useful thyromimetics, providing further details on a subject that has already been widely discussed in recent reviews (25, 26).

## Effects of TH_s_ in the Liver and Central Nervous System

In the liver, THs are actively involved in the regulation of metabolism throughout different pathways (Figure 2). Indeed, THs increase energy expenditure by having a direct effect on ATP consumption in metabolic cycles and membrane permeability as well as by indirect effects on mitochondrial biogenesis and activation (27, 28). In addition, different critical steps in the lipid metabolism are under THs control: cholesterol serum clearance by the low density lipoprotein (LDL) receptor, 3-hydroxy-3-metylgutaryl-coenzyme A reductase, cholesterol biosynthesis and cholesterol 7α-hydroxylase (CYP7A1) (29). Disruption in the normal THs signaling has been also reported to be causative of liver diseases such as non-alcoholic fatty liver disease (NAFLD), a very common disease in Western countries, whereas exogenous T3 administration has been demonstrated to improve the fatty profile in animals with experimental induced NAFLD (30, 31). Finally, neoplastic process in the liver as hepato-carcinoma (HCC) appears to be correlated with THs signaling alterations (32) and some studies demonstrated T3 to be effective in reducing cancer progression and metastasis formation (33). Overall, THs appear significantly beneficial for the liver metabolism. Unfortunately, these positive effects are accompanied by the dangerous effects of thyrotoxicosis, such as tachycardia, muscle wasting, bone impairment, in addition to harmful effects in the brain (3).

Many studies in animal models underlined the importance of TRs for the specific action of THs in the liver [(34); Figure 2]. THs have been demonstrated to regulate 7α-hydroxylase (CYP7A), a crucial liver enzyme in the synthesis of bile acids. TRα1 and TRβ different contribution to this regulation has been studied in TRα1 and TRβ knockout mice treated with 2% dietary cholesterol and T3. The study showed that only TRβ knockout mice were unable to modulate CYP7A1 and that T3 administration was not effective on cholesterol levels in these animals (35), thus suggesting a crucial and specific role of TRβ in liver.

Moreover, this specific role is likely not only dependent on the abundance of TRβ isoform in the liver (which represents the 80% of T3 binding activity in rodent). A distinct zonal expression of the TRs and their respective target genes has been proposed in support of the hepatic target gene specificity by TRs (29). On the other hand, TRα1 is predominant in the myocardium, where it covers 40 −70% of T3 heart-binding capacity, depending on the species (36, 37), and it is responsible for the heart side effects of THs excess.

THs play a very critical role on the central nervous system (CNS) (Figure 2) as it is depicted by the severe effects on neuropsychological, cognitive development, and motor activity of children with congenital hypothyroidism, a condition known as cretinism (38). A perfect synergy between mother's THs actions and normal fetus thyroid development is necessary for neuronal differentiation, migration and proliferation (39). While TRα1 appears to be ubiquitous in CNS regions, TRβ1 is only present in hypothalamus, retina, pituitary gland, and cochlea in later stages of development (40). Indeed, animal studies on TRα1 knockout mice demonstrated delay in the formation of dendrites and axons of GABA-ergic neurons, which are very important for nervous circuits (41). Moreover, THs have been shown to enhance the crucial interaction between Purkinje and granule cells, necessary for the adequate layering of cerebellar cortex: in either TRα1 and TRβ1 knockout mice abnormalities in cerebellum differentiation and function have been described (42). The actions of THs on CNS depend on the availability of the hormones and the controlled transport across blood brain barrier (BBB), which seems to be the preferred route in the adult life. It is not the same in embryo, in which choroid plexus is mainly involved in the transport. Despite the initial belief of a passive diffusion of THs through cell membranes, subsequent data clearly showed that multiple families of THs transporters guarantee a saturable and stereospecific THs uptake (43). The most important carriers in BBB are the monocarboxylate transporter 8 (MCT8) and the Na+ independent organic anion-transporting polypeptide 1C1 (OATP1C1). In mice lacking both MCT8 and OATP1C1, the concentration of THs in brain are significantly affected (44). Transporters of the LAT family LAT1 and LAT2 have been implicated in THs transport across BBB in rodents, whereas LAT1 in human is expressed in brain endothelial cells (45). Once THs have passed BBB, their local availability depends from the activity of the astrocytic type II deiodinase (D2) which converts T4 in T3. T3 is subsequently metabolized into 3,5-diiodothyronine (T2) by type 3 deiodinase (D3) in neurons (39).

It is widely known that THs are required for the proper proceeding of oligodendrocytes differentiation and maturation (46), and a novel role of THs as promoters of remyelination in CNS has been recently suggested, opening a new perspective for the treatment of demyelinating disorders such as multiple sclerosis (MS) (47, 48). In fact, in cells and animal models, THs demonstrated to enhance normal processes of myelination acting on oligodendrocytes development, and a THs deficiency revealed to be associated with a decreasing of the number and function of oligodendrocytes and the expression of myelin basic protein (MBP) in the brain (49). Moreover, THs stimulate the process of remyelination, that occurs after an injury and is triggered by the oligodendrocyte precursor cells (OPCs). These are stem cells that distributed in the CNS and are capable to differentiate in mature myelinating oligodendrocytes (50). This effect is mediated by THs presumably through the induction of neurotrophic expression factors, suppression of apoptosis and OPCs differentiation (51, 52). The full mechanism of THs action on OPCs is not completely understood, but the current hypothesis is that TRβ isoform plays a pivotal role. THs binding to TRβ could act together with RXR activation to promote the expression of a crucial gene for OPCs differentiation, named KLF9 or Kruppel Like Factor 9 (a basic helix loop helix family member) (48). Studies in mice's OPCs showed that the overexpression of TRβ isoform increases the OPCs differentiation, confirming the central role of TRβ (53).

## Thyroid Hormone Receptors Actions and Rationale Design of Selective Thyromimetics

**Figure 4:**
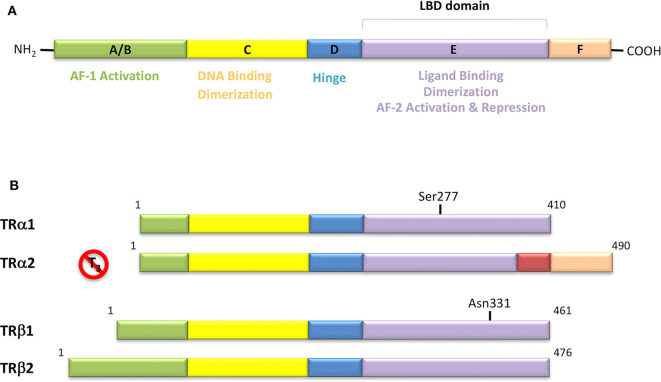
**(A)** Domain organization of Thyroid Hormone Receptors (TRs) and functional regions. The TR is a member of the nuclear receptor (NR) protein family. The NRs share a common modular structure, which contains a variable A/B N-terminal region, a central conserved DNA-binding domain (DBD), a less conserved ligand-binding domain (LBD), and a linker region between the DBD and LBD. The N-terminal region harbors the activation function-1 (AF-1), which is independent of the LBD-ligand interaction. Within the LBD lies the activation function-2 (AF-2), with it referring to the recruitment of transcriptional activators in a ligand-dependent manner. **(B)** Schematic representation of the different isoforms of thyroid hormone receptor. There are two distinct TR genes (alpha and beta) that expressed as two differentially spliced isoforms (α1 and α2, and β1 and β2, respectively). TRα1 and TRβ1 are the predominant ligand binding forms in most tissues. Each receptor isoform conforms to the three-domain structure (NTD, DBD, and LBD) that is typical of NRs. The length of receptors is indicated just above the receptor diagrams. TRα2 cannot bind T3 because it contains a 122-amino acid carboxy terminus that replaces a region in TRα1 that is critical for TH binding.

In the classification of THs effects proposed by Flamant et al., the “type 1 effects” cover the classical genomic actions of THs, which require TRs and direct interaction with DNA (14) TRs act by binding to their cognate TRE, in the promoters of target genes, thereby stimulating or preventing transcriptional activity (Figure 1). TRs can bind as monomers, homodimers, and heterodimers to TREs (54). TRs have been shown to form heterodimers with several TR auxiliary proteins (TRAPs) that increase TR binding to TREs. Among them, the retinoid X receptors (RXRs) represent the predominant form of TRAPs and thus play a fundamental role in TH-mediated transcription (54). The regulation of transcription by TH involves various regulatory proteins, namely coactivators (i.e., SRC-1, CBP/p300) and corepressors (i.e., SMRT, NCoR), that bind to the receptor–DNA complexes and either promote or repress transcription, respectively (Figure 1). The interaction site of coactivators with nuclear hormone receptors involves a recognition motif characterized by the amino acid sequence (LXXLL) (55). TRs have a well-defined domain organization, common to all nuclear hormone receptors, consisting of an amino-terminal A/B domain, a DNA binding domain (DBD), a hinge region that connects the DBD to the Ligand Binding Domain (LBD), and the C-terminal of the LBD (Figure 4). TRs have a compact LBD, produced by a three-layer antiparallel α-helical sandwich formed by 12 α-helices. The dynamic of the AF-2 region located in helix 12 of the LBD plays a critical role in regulating the interaction of the receptor with coactivators or corepressors following agonist or antagonist binding to the LBD, respectively (56–58). Notably, this compact helical organization generates a “wedge-shaped” structural arrangement into which the hormone binds resulting deeply buried within the receptor (59).

TRs are also involved in “type 2 effects” of THs (14), without direct interaction with DNA. Consistent data are available suggesting that TRs can modulate chromatin by a protein-protein interaction, likely with other transcription factors (60), thus influencing epigenome. TRs have been also demonstrated to be active in the cytoplasm, beyond nuclear action and without interacting with DNA, participating in intracellular signaling cascades (type 3 effects of THs). It is noteworthy that some THs effects exist that seem to be independent from TRs, with different hypothesized mechanisms (use of integrin αVβ3 as membrane receptor, direct allosteric regulation of metabolic enzymes) (14).

TRs are obviously the natural target of any drug with thyromimetic function. The LBD represents the most relevant domain when designing putative thyromimetics (61). The selectivity of the THs effect on metabolism and liver is due to a crucial difference in the crystal structure of the TRα1 and TRβ: one residue in the binding cavity i.e., Asn 331 in TRβ and Ser 277 in TRα1 [(59); Figure 4]. This understanding led to the idea of selective thyromimetics. In 1998 a novel compound named GC-1 (Sobetirome) (Figure 3) was synthesized, by Chiellini et al., which was capable to bind TRβ with the same affinity as T3, but TRα1 with 10-fold lower affinity (62). The structural characteristics responsible for the selectivity of GC-1 are the presence of the oxyacetic acid chain, replacing amino acid side chain, that enhances polar interaction with Arginine residues in the TRβ pocket, and the presence of a diphenylmethane scaffold where the 3,5 di-methyl substitutions allow the ligand to be locked in the active perpendicular conformation with respect to the terminal phenyl ring, while establishing many van der Waals interactions with the lipophilic side chains of numerous amino acid residues present in the ligand binding cavity (LBC). Other profitable lipophilic interactions were also established by the iso-propyl substituent at position 3′ of the outer ring (59). GC-1 demonstrated many beneficial effects on hepatic metabolism, in the absence of heart side effects, and during last years a number of selective thyromimetics have been produced (63, 64), either with a classical thyronine based phenoxyphenyl-structure, including KB141 (10) and KB2115 (65) or with a Sobetirome-like diphenylmethane scaffold, as summarized in the following section (Figure 3).

## TRβ -Selective Thyromimetics Showing a Thyronine Based Aryloxyphenyl-Structure

### Eprotirome (KB2115)

(3-[[3,5-dibromo-4-[4-hydroxy-3-(1-methylethyl)-phenoxy]-phenyl]-amino]-3-oxopropanoic acid) (Figure 3) is a TH analog that has modestly higher affinity for the TRβ isoform compared with its affinity for the TRα isoform and displays hepatic-selective uptake (65). Data collected from initial short-term studies testing Eprotirome for the treatment of hyperlipidemia in humans appeared very promising. Administration of Eprotirome was found to reduce serum cholesterol (up to 40%) and ApoB without producing undesirable side effects. Notably, no cardiac toxicity was observed [(66); Table 1]. Despite the promising activity on LDL cholesterol, triglyceride, ApoB, and Lp(a) lipoprotein reduction observed in subsequent clinical studies, a Phase 3 trial was terminated because alterations of dog cartilage were observed following chronic treatment (NCT01410383). In addition, reduced T4 levels, as well as liver toxicity, occurred in homozygous patients affected by familiar hypercholesterolemia, after only 6 weeks of treatment with 50 or 100 μg of Eprotirome (69).

**Table 1:** Effects of TRβ-selective thyromimetics in preclinical studies and human trials.

**Thyromimetic**	**Structure**	**Beneficial effects**	**Side effects/clinical trials**
GC-1 (Sobetirome) *QuatRx*	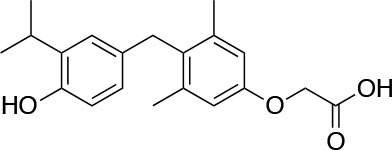	↓LDL cholesterol ↓Serum triglycerides ↓Lipoprotein a [Lp(a)] [Scanlan (67); Hartley (68)]	No adverse side effects associated with excess TH Terminated after Phase 1
Eprotirome (KB2115) *KaroBio*	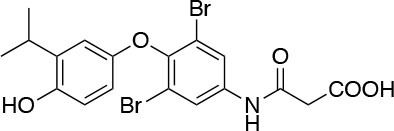	↓Total cholesterol ↓LDL cholesterol ↓Lp(a) ↓triglycerides [Berkenstam et al. (66); Sjouke et al. (69)]	Upregulation of LDL receptors and cardio-vascular risks Liver toxicity; increase of AST and ALT levels Cartilage Defect in Dogs Terminated during Phase 3
Resmetirom (MGL-3196) *Madrigal Pharmaceutical*	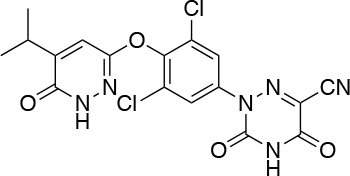	↓Hepatic fat in patients with NASH [Kelly et al. (70); Harrison et al. (71)]	No adverse side effects after Phase 2 clinical trails Phase 3 in progress (NCT03900429)
VK2809 (MB08711) *Viking Therapeutics*	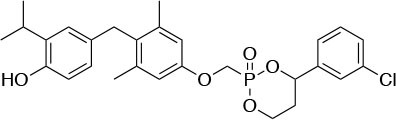	↓LDL-C ↓Liver fat content in patient with NAFLD [Erion et al. (72)]	Almost negligible side effects reported Phase 2b in progress (NCT4173065)
Sob-AM2 *Llama Therapeutics*	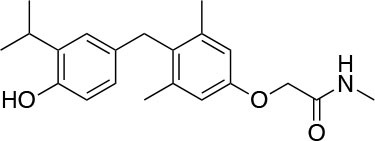	↑Myelin repair [Hartley et al. (73)]	Preclinical testing
IS25 *I.S.D.D. srl*	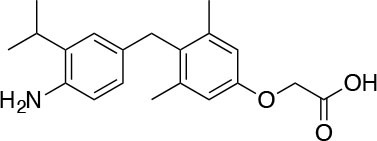	↑Lipolysis ↓Triglycerides ↑Hepatocyte proliferation [Runfola et al. (74); Perra et al. (75)]	Preclinical testing
TG68 *I.S.D.D. srl*	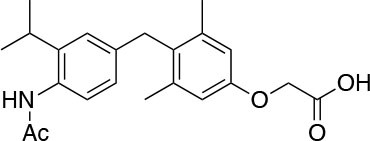	↑Lipolysis ↓Triglycerides ↑Hepatocyte proliferation [Runfola et al. (74); Perra et al. (75)]	Preclinical testing

### Resmetirom (MGL-3196)

2-[3,5-Dichloro-4-(5-isopropyl-6-oxo-1,6-dihydropyridazin-3-yloxy) phenyl]-3,5-dioxo-2,3,4,5-tetrahydro[1,2,4]triazine-6-carbonitrile (Figure 3) is a recently developed liver-directed and markedly TRβ-selective agonist (EC50s = 0.21 and 3.74 μM for TRβ and TRα, respectively) (70). Preclinical, toxicology, Phase 1, and Phase 2 clinical data suggest Resmetirom has great potential for the treatment of non-alcoholic steatohepatitis (NASH), non-alcoholic fatty liver disease (NAFLD), and associated dyslipidemias (Table 1). Notably, Resmetirom has shown neither suppression of the central thyroid axis nor TRα effects on heart rate or bone, and it reduces elevated liver enzymes in NASH patients. On the basis of positive Phase 2 clinical trial results in patients with NASH (71), which demonstrated significant effects in reduction of hepatic fat and markers of inflammation and fibrosis, as compared to placebo, Madrigal Pharmaceuticals recently started a Phase 3 multinational, double-blind, randomized, placebo-controlled clinical trial of Resmetirom in patients with NASH and liver fibrosis (ClinicalTrials.gov Identifier: NCT03900429).

## Sobetirome and Novel Diphenylmethane Structure Based Thyromimetics

### Sobetirome (GC-1)

(3,5-Dimethyl-4 (4′-hydroxy-3′-isopropylbenzyl) phenoxy acetic acid) (Figure 3) differs from T3 for the presence of three hydrocarbon residues in place of the iodine atoms, the methylene linkage between the two phenyl rings, and the substitution of the 1-aminopropionic acid with oxyacetic acid. When it was first synthesized and tested, in the late 1990s, the selectivity for TRβ isoform was an unexpected finding (67). At that time, this selectivity was unequivocally proved by running studies in a model of *Xenopus laevis* tadpole growth. In the frog, metamorphosis occurs as a result of gene expression cascades induced by TH secreted from the developing tadpole's thyroid gland. Xenopus TRα is expressed early in development, long before the larval tadpole have a functional thyroid gland, whereas much lower levels of Xenopus TRβ mRNA are detectable prior to metamorphosis. At metamorphosis, TRβ is strongly induced by TH in larval tissues that will die and resorb such as the tail. In this model, treatment of premetamorphic tadpoles with GC-1 actively stimulated tail and gill resorption, that are a TRβ dependent mechanism, while poorly inducing limb bud development, typically TRα mediated. Notably, the effectiveness of GC-1 in inducing tail resorption and tail gene expression correlates with increasing TRβ levels (76).

GC-1 beneficial effects on lipid profile has been shown firstly in hypothyroid mice and rats with hypercholesterolemia, where it enhanced the lowering of cholesterol and triglycerides, without important side effects in cardiomyocytes and muscle cells (77). GC-1 has direct effects in promoting the so called “reverse cholesterol transport” which describes the clearance of atherogenic cholesterol from extrahepatic tissues to the liver, by increasing the activity of the high density lipoprotein (HDL) and LDL receptor on hepatocytes and by the stimulation of CYP7A1 enzyme (8). GC-1 is also active in preventing atherosclerosis by lowering the levels of cholesteryl esters in the vasculature, as demonstrated in a model of Apolipoprotein E (Apo-E) deficient mice (56, 78), and is effective in stimulating the brown adipose tissue (BAT) thermogenesis, thus reducing fat mass [(79); Table 1].

Although a large body of evidence supports the efficacy of GC-1 as a potent and hepatoselective lipid- lowering agent, in 2008 it was terminated by QuatrX after completed one Phase 1 clinical trial due to lack of funding.

Due to the impact of liver chronic diseases in public health and based on previous findings with T3, GC-1 was also tested in models of NAFLD, HCC, and hepatectomy. In a rodent model of NAFLD, obtained by choline devoid methionine deficient (CMD) diet, GC-1 showed to be effective in preventing or promoting the improvement of the induced hepatic steatosis (80). This and other evidences in the literature confirmed a possible role of GC-1 as antisteatogenic compound, even though some authors advice caution because hyperglycemia and insulin resistance have been observed in some of the experiments (81). An interesting effect of GC-1 has been demonstrated in hepatic neoplastic proliferation: in a rat model of HCC the treatment with GC-1 for 2 weeks caused the regression of pre-neoplastic nodules and the induction of markers of hepatic differentiation (82). The finding that TRβ isoform is downregulated in hepatic neoplasms is another indirect evidence that restoring THs molecular effects, could be of benefit against tumor progression.

Finally, a promising field of investigation for GC-1 is its action as potent inducer of hepatic proliferation: in rodent models of partial hepatectomy, the pre-treatment with GC-1 led to a significant increase in hepatocytes proliferation, without substantial side-effects (83). The mechanism underlying this effect seems to be mediated, at least in part, by the activation of β-catenin pathway, since the potent effect on proliferation is lost in liver specific β-catenin knockout mice (83).

In respect to the action of GC-1 in the CNS, as we already mentioned, GC-1 was tested in an *in-vitro* system, that allowed to measure quantitatively and qualitatively oligodendrogenesis. In this model, GC-1 was found to promote the differentiation of the OPCs in both in murine and human cells. Moreover, during the oligodendrogenesis promoted by GC-1, TRβ1 was shown to be upregulated. Finally, the same group demonstrated that it was possible to show and monitor the GC-1 induced oligodendrogenesis in the corpus callosum, occipital cortex and optic nerve using an *in-vivo* transgenic mouse model (73, 84).

#### VK2809 (MB07811)

Recently, a new strategy to overcome the detrimental effects related to TR activation in extrahepatic tissues has been the development of liver targeting “TR agonist prodrugs.” VK2809 is the most promising of the prodrug candidates and was originally developed by Metabasis Therapeutics, Inc., under the name of MB07811 (2R,4S)-4-(3-chlorophenyl)-2-[(3,5-dimethyl-4-(4′-hydroxy-3′-isopropylbenzyl) phenoxy)methyl]-2-oxido-[1–3]-dioxaphosphonane (24, 72). Notably, from a structural point of view, this new generation of Hep-Direct prodrugs are small aryl-substituted cyclic prodrugs (Figure 3) that are able to generate the active drug after oxidation of the benzylic methine proton produced by the cytochrome P450 (CYP) isoenzyme CYP3A. This leads to the irreversible opening of the ring and the formation of phosphonate, that binds to TRs (72). Indeed, pharmacokinetic studies in rats demonstrated that the prodrug (MB07811) is exposed to first-pass hepatic extraction and after cleavage generates the negatively charged 3,5-dimethyl-4-(4′-hydroxy-3′-isopropylbenzyl)phenoxy)methylphosphonic acid (MB07344), which has a very low tissue distribution and rapidly undergoes to elimination in the bile. The activated form has a more pronounced affinity for TRβ (Ki = 3 nM) than for TRα (Ki = 35 nM), and preclinical studies showed that when administered in hyperlipidemia and normal rodent models, MB07811 was able to produce a significant reduction of total plasma cholesterol and hepatic and plasma triglycerides [(72); Table 1]. Subsequently, MB07811 was used in human trials. Notably, a Phase 1 multiple-ascending dose study in patients with mild hypercholesterolemia, revealed that administration of VK2809 (MB07811) was able to induce a significant reduction in LDL-C, triglycerides and atherogenic proteins. In addition, the results of a Phase 2 trial for the treatment of NAFLD and elevated LDL-C, showed that the administration of VK2809 significantly reduced LDL-C and liver fat content as compared to placebo treated patients. Notably, in both trials VK2809 resulted also to be safe and well-tolerated. At last, few months ago Viking Therapeutics announced the initiation of a Phase 2b clinical trial of VK2809, in patients with biopsy-confirmed NASH (ClinicalTrials.gov identifier: NCT4173065). Given VK2809's promising initial data on both liver fat and plasma lipids, this novel diphenylmethane prodrug may quickly move forward to reach the market as a new drug for the treatment of a large variety of lipid related liver pathologies, including NASH.

#### Sob-AM2: A CNS-Selective Prodrug of Sobetirome

A novel field of interest is the possible use of selective thyromimetics in demyelinating diseases, such as multiple sclerosis (MS), that have poor prognosis and are in need of new therapeutic approaches. As reported, TRβ isoform seems to play a pivotal role in the remyelination process, that is induced by THs. Consistently, GC-1 (Sobetirome) has been proved to distribute to the CNS *in vivo* and to induce the differentiation of OPCs in human and rodent *in vitro* models (84). However, the presence of a carboxylate negatively charged group in the structure makes difficult for GC-1 to cross the blood brain barrier. More recently, with the aim to combine the TRβ selectivity of GC-1 with a better brain tissue selectivity and capacity to cross the blood brain barrier, new prodrugs of GC-1, named Sobetiramides, have been produced by masking the carboxylate group with easily cleavable protecting groups (85).

### Sob-AM2

Sob-AM2, the methyl amide derivative of Sobetirome (Figure 3), is an optimized prodrug that significantly increases delivery of Sobetirome to the brain while concomitantly decreasing blood and peripheral organ exposure to the active drug (85). Notably, in mice lacking Mct8 and Dio2, Sob-AM2 treatment has been shown to increase Sobetirome brain content and exert central and peripheral thyromimetic actions by modulating the expression of T3-dependent genes (86). Since a concomitant decrease of circulating T4 and T3 was also observed in the same animal model, the potential for Sob-AM2 to address the cerebral hypothyroidism and the peripheral hyperthyroidism characteristic of MCT8 deficiency has been consistently suggested (86). Using iCKO-Myrf mice, a recently developed genetic mouse model of demyelination and remyelination, where demyelination was induced by tamoxifen treatment of adult *Myrf*^*fl*/*fl*^; *Plp1-CreERT* mice Hartley et al. (73) and Koenning et al. (87) demonstrated that chronic treatment with Sobetirome (80 μg/kg/d, p.o.) or Sob-AM2 (84 μg/kg/d, p.o.) leads to significant improvement in both clinical signs and remyelination, as assessed by using motor function tests, histology, and MRI techniques (Table 1). Notably, Sob-AM2 is selectively hydrolysed by the fatty acid amide hydrolase (FAAH), a crucial enzyme, highly expressed in the CNS (85, 88, 89), and efficiently converted to Sobetirome. Therefore, when administered systemically, Sob-AM2 leads to increased CNS distribution and decreased peripheral exposure of the active drug Sobetirome (85). However, after oral administration of Sob-AM2 in the iCKO-Myrf model, a significant conversion to Sobetirome was observed to occur in the gastrointestinal tract, conducing to increased Sobetirome and decreased Sob-AM2 concentration in the blood. Furthermore, Sob-AM2 displayed an oral bioavailability ~5-fold lower than that of Sobetirome. As a consequence of these two issues, orally dosed Sob-AM2 resulted in only a modest (~2- to 4-fold) increase of CNS Sobetirome level, as compared with the same oral dose of Sobetirome (85, 90). Consistently, only a moderate improvement in rotarod performance was observed in iCKO-Myrf mice treated with 84 μg/kg/d (p.o.) Sob-AM2 as compared to mice receiving an equivalent dose of Sobetirome. In the same study, it was observed that chronic treatment with TH (hyperthyroidism) inhibited the endogenous myelin repair and aggravated disease. Therefore, these results further underscore the need for selective TH action to promote remyelination in the CNS. To date, Sobetirome, Sob-AM2 (85), and other recently developed Sobetirome derivatives (90–92) are the only thyromimetics reported to distribute to the CNS from systemic administration, and might represent the first class of TH agonists that can be subjected to clinical evaluation in demyelinating diseases such as MS.

Recently, Sobetirome has been proposed for the treatment of X-linked adrenoleukodystrophy (ALD), a rare congenital disease due to mutations in the ALD protein, characterized by adrenal insufficiency and central nervous system (CNS) demyelination. No specific pharmacological therapy is available for ALD. All patients with X-ALD have the biochemical abnormality of elevated blood and tissue levels of very long chain fatty acids (VLCFAs), namely saturated fatty acids with ≥22 carbon atoms. Notably, in a transgenic mouse model of ALD, Sobetirome administration reduced the brain and adrenal content of very-long-chain fatty acids (68). Based on these observations, a clinical trial with sobetirome in X-linked ALD was posted in the NIH database (NCT01787578). Unfortunately, in March 2019 NeuroVia, Inc. withdrew a phase I/II trial prior to enrolment due to no funding in Adrenoleukodystrophy (in children, in adolescents) in Australia, United Kingdom, Chile (PO) (NCT03196765).

#### Novel 4′-Amino-Benzyl-Phenoxyacetic Acid Thyromimetics: IS25 and TG68

Novel halogen free TRβ selective agonist IS25 and its pro-drug TG68 (Figure 3) have been recently identified (74, 93). Notably, both compounds initially exposed to *in vitro* analysis of cytotoxicity and ADME-Tox/off-target liability revealed a convincing lack of toxicity (74, 94), supporting their progression in the drug discovery process. *In vitro* investigation proved that both compounds were able to reduce lipid accumulation in human hepatoma HepG2 cells by promoting lipolysis with a potency comparable to that of T3, convincingly showing that derivative TG68 was capable of efficiently deliver the corresponding parent compound IS25 (Table 1). Moreover, western blot analysis showed that the decreasing of lipid accumulation observed in HepG2 cells after treatment with both compounds was related to the stimulation of AMPK phosphorylation, concomitantly leading to the phosphorylation and consequent inactivation of acetyl coenzyme A carboxylase (ACC), the major regulator of fatty acids synthesis (74). In addition, *in vivo* studies confirmed the lipolytic action and the apparent lack of toxicity that had already been observed for both compounds with the *in vitro* experiments. Indeed, reduced triglyceride levels and no liver injury, as assayed by serum levels of ALT, AST, and bilirubin, combined to the absence of cardiac hypertrophy, were observed in F344 rats exposed to a sub-chronic treatment with the two novel TRβ-agonists (74). Nevertheless, additional *in vivo* studies will be required in order to validate the therapeutic potential of both compounds in fatty liver dysfunctions, including NAFLD and NAFL.

It is widely known that thyroid hormone T3 represents a potent hepatomitogen, and its mitogenic effect seems to be mediated by TRβ (95). Therefore, TRβ-selective thyromimetics devoid of relevant side effects might find a possible use in regenerative medicine (83, 96). Even though still at a preliminary level, ongoing studies have revealed that in F344 rats a sub-chronic treatment with the two novel TRβ-agonists, namely IS25 and TG68, induced hepatocyte proliferation in the absence of any sign of hepatic toxicity and cardiac hypertrophy (75). Notably, hepatocyte proliferation appears to be associated with activation of TR-target genes, such as Dio1 and Spot14, suggesting that the mitogenic effect of these drugs may be due to binding and activation of TRβ. Importantly, the liver proliferative response induced by the two TRβ agonists was not associated with liver damage, as assessed by biochemical determination of serum transaminases and by immunostaining for Caspase-3, but it was the result of a direct effect of these drugs enabling quiescent hepatocytes to re-entry into the cell cycle (75). Hence these newly developed agents may have a significant clinical application for hepatic regenerative therapies or other surgical procedures.

#### Glucagon/t3 Hybrid: A Promising Alternative to Isoform-Selective Thyromimetics

A new perspective for the therapy of metabolic syndrome has recently emerged from the work of Finan et al. (97). The authors generated a Glucagon/T3 hybrid molecule to release both the beneficial anti-lipid effects of glucagon and the energy expending effects of T3. The most remarkable aspect of this new Glucagon/T3 hybrid is the ability to confer key beneficial metabolic effects of each component, while avoiding the negative side effects observed for each hormone separately. Indeed, the use of Glucagon/T3 in obese mice ameliorates serum dyslipidemia, diminishes adipose mass, reverses NASH, reduces atherosclerotic plaque accumulation, and improves glucose metabolism, while avoiding thyrotoxicosis and the diabetogenic effects of glucagon. Even though additional investigations will be required for the advancement of this compound to clinical trials, the engineered Glucagon/T3 co-agonism holds promise as an alternative to isoform-selective thyromimetics.

## Conclusions

TRβ is the predominant isoform of TR in the liver and therefore is primarily responsible for the reduction of cholesterol levels, whereas the adverse effects on heart and bone are mainly connected to TRα. Thus, in the development of clinically useful drug candidates, much effort has been directed toward the design of compounds with isomer-specific activity related to the structure of thyroid hormones, i.e., thyromimetics that are liver and/or TRβ selective. Among the several TRβ-selective T3 analogs generated in the past two decades, Sobetirome (GC-1), Eprotirome (KB2115), Resmetirom (MGL-3196) and the Hep-Direct prodrug VK2809 (MB07811) have demonstrated to mimic most of the beneficial effects of T3, in the absence of adverse side effects. For example, Sobetirome and Eprotirome entered human clinical trials for dyslipidemia, showing promising results in the absence of deleterious effects typically associated with hyperthyroidism [for reviews, see (63, 98)]. Unfortunately, no Phase 2 trials for GC-1 have been performed and a Phase 3 trial with KB2115 was terminated due to harmful effects on cartilage observed in canines. In spite of this disappointing result, Resmetirom (MGL-3196) successfully completed Phase 2 trials showing to potently reduce liver fat content after 12 and 36 weeks of treatment in patients with NASH in the absence of significant side effects, and was recently advanced to Phase 3 trials. Liver and TRβ-selective agonist VK2809 also showed positive results from a Phase 2 trial in patients with hypercholesterolemia and NAFLD, and is currently being evaluated in a Phase 2b clinical trial in patients with biopsy-confirmed NASH. Taken together, all these findings suggest that modern thyromimetics hold promise for dyslipidemia.

Due to their encouraging effects in metabolism control and CNS pathologies, thyromimetics have arisen much interest in the field of medical research; however, the combination of receptor and organ selectivity is now a fundamental requirement for the development of novel compounds.

Sob-AM2, a CNS-selective prodrug of Sobetirome, has been shown to promote brain and spinal cord myelin repair. This effect was associated with a significant improvement in neurological clinical signs that stem from CNS demyelination. Notably, at the present time there is still a lack of approved treatments for MS that stimulate myelin repair, therefore Sob-AM2 holds promise as a effective drug candidate for the treatment of demyelinating disorders, such as MS.

During a drug discovery investigation aimed at identifying novel TRβ-selective agonists, the application of a comprehensive panel of ADME-Toxicity assays enabled the selection of analog IS25 and its prodrug TG68 as the best candidates to progress along the drug discovery pipeline. Their lipid lowering properties have been well-documented by both *in vitro* and *in vivo* assays. Importantly, preliminary results from *in vivo* studies indicate that they are able to potently produce hepatocyte proliferation without causing liver damage or cardiac and renal hypertrophy, classically associated to the treatment with THs. Therefore, these compounds may result clinically useful in the context of regenerative medicine, for the treatment of conditions where a rapid liver cell proliferation is required or when liver regenerative capacity is impaired.

In conclusion, last decade has seen a reawakening of interest in developing TRβ selective TH analogs, and at the present there is renewed hope for novel tissue- and/or isoform-selective thyromimetics to find application in therapy for the treatment of dyslipidemia and liver-related life threatening conditions, as well as for disabling diseases of the CNS, such as MS. Even though still at its infancy as a therapeutic tool, the engineered Glucagon/T3 co-agonism opens a new scenario in the search of a pleiotropic approach to the treatment of diseases that would benefit of T3 tissue and isoform selective actions. It is arguable that in the near future the use of this “combinatorial chemistry” technology will see a further expansion to maximize the benefits and minimize the risks of each component as compared to individual forms of administration.

## Author Contributions

GC and SR had the idea of this review, wrote most of the paper, and organized final draft. FS and SS wrote the initial section of the paper and prepared the figures. MR edited and revised the paper. All authors revised the final manuscript and approved the final version.

## Conflict of Interest

The authors declare that the research was conducted in the absence of any commercial or financial relationships that could be construed as a potential conflict of interest.

## Abbreviations

ACC: acetyl coenzyme A carboxylase
Apo-E: Apolipoprotein E
BAT: brown adipose tissue
CMD: choline devoid methionine deficient
CNS: central nervous system
CYP: cytochrome P450
CYP7A1: 7 α-hydroxylase
D3: type 3 iodothyronine deiodinase
DBD: DNA binding domain
FAAH: fatty acid amide hydrolase
HAT: histone acetyltransferase
HCC: hepato-carcinoma
HDAc: Histone Deacetylase
HDL: high density lipoprotein
LAT1: L-type amino acid transporter1
LBC: ligand binding cavity
LBD: ligand binding domain
LDL: low density lipoprotein
KLF9: Kruppel Like Factor 9
MBP: myelin basic protein
MS: multiple sclerosis
NAFLD: non-alcoholic fatty liver disease
NASH: non-alcoholic steatohepatitis
OPCs: oligodendrocyte precursor cells
RXRs: retinoid X receptors
T3: 3,3′,5-triiodo-L-thyronine
T4: 3,3′,5,5′-tetraiodo-L-thyronine
THA: thyroid hormone axis
THs: Thyroid hormones
TRAPs: TR auxiliary proteins
TREs: TH response elements
TRs: THs receptors.
